# Corn Stover Replacement with *Quercus rugosa* Woody Biomass: Growth Performance, Carcass Characteristics, and Meat Quality of Finishing Lambs

**DOI:** 10.3390/ani16213339

**Published:** 2026-10-23

**Authors:** Octavio Carrillo-Muro, Alejandro Rivera-Villegas, Pedro Hernández-Briano, Carlos Aurelio Medina-Flores, Marco Antonio López-Carlos, Carlos Fernando Aréchiga-Flores, Francisco J. Gutiérrez-Piña, Oliver Yaotzin Sánchez-Barbosa, Daniel Rodríguez-Cordero, Alejandro Plascencia

**Affiliations:** 1 Unidad Académica de Medicina Veterinaria y Zootecnia, Universidad Autónoma de Zacatecas, General Enrique Estrada 98500, Zacatecas, Mexico; alejandro.rivera@uaz.edu.mx (A.R.-V.); pedro.hernandez@uaz.edu.mx (P.H.-B.); carlosmedina@uaz.edu.mx (C.A.M.-F.); lopcarmarco@uaz.edu.mx (M.A.L.-C.); arechiga@uaz.edu.mx (C.F.A.-F.); fpina@uaz.edu.mx (F.J.G.-P.); oliver.sanchez@uaz.edu.mx (O.Y.S.-B.); 2 Instituto de Investigaciones en Ciencias Veterinarias, Universidad Autónoma de Baja California, Mexicali 21100, Baja California, Mexico; daniel.rodriguez64@uabc.edu.mx; 3 Facultad de Medicina Veterinaria y Zootecnia, Universidad Autónoma de Sinaloa, Culiacán 80260, Sinaloa, Mexico; alejandro.plascencia@uabc.edu.mx

**Keywords:** alternative feed resources, economic evaluation, fiber replacement, finishing lambs, meat quality, *Quercus rugosa*

## Abstract

This study evaluated the growth performance, blood metabolites, carcass characteristics, meat quality, and economic responses of finishing lambs fed diets in which condensed tannin-containing woody biomass (CTWB) from *Quercus rugosa* partially or completely replaced corn stover (CS) in the fibrous fraction. Forty-eight intact male Dorper × Katahdin lambs (initial body weight: 33.01 ± 1.23 kg), housed in 24 pens with two lambs per pen, received diets containing 0, 8, 16, or 24% CTWB (CTWB-0, CTWB-8, CTWB-16, and CTWB-24, respectively) for 42 d during 2013–2014. The 24% inclusion level replaced all the CS and supplied a calculated contribution of 6.29 g of condensed tannins/kg dry matter (DM) from CTWB. Dry-matter intake (DMI) was greater with CTWB-24 than with CTWB-0 and CTWB-16 (1.34 vs. 1.17 and 1.18 kg DM/d, respectively; *p* = 0.021). Growth and gain-to-feed ratio did not differ among treatments (*p* ≥ 0.240). Blood urea nitrogen concentrations were lower with CTWB-24 than with CTWB-8 (*p* = 0.014). Carcass weights did not differ (*p* ≥ 0.779), nor did cooling loss or dressing percentage (*p* = 0.459 and 0.060, respectively). An overall treatment effect was observed for muscle a* (*p* = 0.049), without differences between individual treatments; dorsal fat b* did not differ (*p* = 0.051), nor did water loss, pH, or shear-force measurements (*p* ≥ 0.103). Calculated diet cost decreased from 0.301 to 0.282 USD/kg DM between CTWB-0 and CTWB-24, but this reduction did not translate into lower total feed cost per lamb (*p* = 0.150). Carcass income and income over feed cost (IOFC) also did not differ (*p* ≥ 0.895); IOFC was 205.56 and 202.21 USD/lamb, respectively. Complete replacement of CS with 24% CTWB was accompanied by greater DMI, while no significant differences were detected in growth, carcass weight, or IOFC in this trial with six pens assigned per treatment. These findings do not establish equivalence among diets, and the limited replication means that biologically relevant differences cannot be ruled out.

## Simple Summary

In high-energy finishing diets for lambs, the source of dietary fiber can influence feed intake, growth, and carcass value. We evaluated woody biomass from *Quercus rugosa* as a replacement for corn stover. A diet containing 24% biomass completely replaced the corn stover during a 42 d feeding period. Lambs fed this diet consumed more feed, while growth, feed efficiency, and carcass weight did not differ among diets. Carcass weight loss during chilling did not differ among diets. Most meat-quality measurements and the economic indicators also did not differ. The lower calculated cost per kilogram of diet did not translate into lower feeding costs per lamb. These findings are exploratory: the limited number of pens means that potentially important differences between diets cannot be ruled out, and similar performance has not been established.

## 1. Introduction

In intensive finishing systems, a fibrous fraction in high-energy diets helps maintain suitable ruminal conditions, while the amount and source of fiber can affect feed intake and growth performance [1,2]. This has encouraged interest in alternative fiber sources that could replace conventional ingredients, such as corn stover (CS), without compromising animal performance [2]. Corn stover has been used as the fibrous ingredient in Mexican lamb finishing diets [3] and was selected here as the reference crop-residue fiber source. Trees and shrubs are potential sources of biomass because they provide fiber, crude protein (CP), and secondary compounds such as condensed tannins (CT) [4].

Condensed tannin-containing woody biomass (CTWB) has these characteristics, although its value as a feed ingredient depends on the material used and its inclusion level. Its assessment as a replacement for CS requires considering its nutrient composition together with its secondary compounds. *Quercus* foliage can also contain hydrolysable tannins [5]. These compounds and non-tannin phenolics were not quantified in the biomass used here; consequently, CT content does not describe its complete phenolic composition. Among these compounds, CT can influence ruminal fermentation and nutrient utilization, with responses varying according to their dietary concentration, origin, structure, and reactivity [6,7,8]. Moderate concentrations have been associated with reduced ruminal CP degradation and improved nitrogen (N) utilization [6,7,9], as well as changes in certain meat characteristics [10]. However, some tannin sources or higher concentrations can reduce dry-matter intake (DMI), growth performance, and apparent CP digestibility [11,12]. These responses also depend on feeding duration and dietary context [13,14]. Thus, CT are one characteristic relevant to the assessment of CTWB, while its suitability as a fiber source must be evaluated through the animal response to the whole ingredient.

Among potential sources of CTWB, the genus *Quercus* is widely distributed across the Northern Hemisphere and is an important component of temperate forests [15]. Its foliage and acorns have been used as feed resources for sheep and goats in several regions [4,16,17]. Mexico is an important center of *Quercus* diversity [18]. One of its species, *Quercus rugosa*, produces biomass during natural defoliation between March and April, with CT concentrations of approximately 22–26 g/kg dry matter (DM) and litter accumulation of up to 616 kg DM/ha [19]. The biomass used here came from that collection and had a reported CT concentration of 26.2 g/kg DM (2.62% DM), slightly above the rounded upper limit of the approximate range. Leaves, foliage, and acorns from several *Quercus* species have shown nutritional potential for ruminants [20,21,22]. In particular, the *Q. rugosa* biomass characterized by Carrillo-Muro et al. [19] contained 3.9% CP, 58.2% neutral detergent fiber (NDF), 1.5% ether extract, and 6.0% ash on a DM basis. Additionally, *Q. resinosa* foliage has shown adequate digestibility and acceptance by lambs [23]. In the price scenario used for this study, CS was valued at 0.11146 USD/kg DM, compared with 0.03450 USD/kg DM for the CTWB evaluated, providing an economic rationale for assessing CS replacement. However, litter accumulation is not equivalent to recoverable feed supply: commercial feasibility depends on seasonal and site variation, collection, processing, transport, storage, and the amount retained for ecological functions. Sustained commercial availability was not evaluated in this study.

Despite this evidence, research on *Quercus* feed resources has focused mainly on their composition, digestibility, preference, and the use of foliage or acorns. A recent study evaluated *Q. rugosa* CTWB as a replacement for CS in prospective breeding Rambouillet lambs [24], but its different production stage, forage proportion, and inclusion levels limit extrapolation to finishing diets. Less is known about using *Q. rugosa* CTWB to progressively replace CS in finishing diets, particularly the growth, carcass, meat quality, and economic responses when CS is partially or completely removed from the formulation. This substitution also changes the fiber source, the dietary concentrations of CP and NDF, and the calculated contribution of CT. Responses must therefore be interpreted in relation to the complete diet rather than attributed to a single component of CTWB.

The objective of this study was to compare finishing lamb diets containing 0, 8, 16, or 24% *Q. rugosa* CTWB, representing partial or complete replacement of CS within a fibrous fraction comprising 24% of dietary DM. To assess its use as an alternative fiber source, we measured growth performance, blood metabolites, ultrasound traits, non-carcass components, carcass characteristics, meat quality, and economic outcomes. The exploratory analysis assessed treatment differences; complete replacement described the removal of CS from the formulation, without implying equivalence or non-inferiority.

## 2. Materials and Methods

### 2.1. Study Site, Ethical Approval, and General Procedures

The finishing trial was conducted from 25 November 2013 to 5 January 2014 at the Small Ruminant Experimental Center of the Unidad Académica de Medicina Veterinaria y Zootecnia, Universidad Autónoma de Zacatecas (UAMVZ-UAZ), in north-central Mexico (22°58′49.09″ N, 102°42′57.26″ W; 2157 m above sea level). The CTWB used in the trial came from *Q. rugosa* vegetation collected in 2013 from a natural forest at Rancho Arroyo de Carrillo, Tequila, Jalisco, Mexico. Ingredients and diets were chemically analyzed at the Animal Nutrition Laboratory, whereas carcass and meat-quality evaluations were performed at the Meat Science and Technology Laboratory of UAMVZ-UAZ.

Animal handling, transport, and slaughter were conducted in accordance with Mexican standards [25,26,27,28]. The experimental protocol was reviewed and approved by the Institutional Committee on Bioethics and Animal Welfare of UAMVZ-UAZ (Protocol No. 2013/05/18; approved 19 May 2013). At enrollment, the lambs showed no evident clinical signs of disease. No inclusion or exclusion criteria were established beyond the animal characteristics described for the study. Trained personnel monitored their health, feed intake, and general welfare daily. No lamb experienced an adverse event requiring removal; all completed the trial and were included in the analyses.

### 2.2. Condensed Tannin-Containing Woody Biomass Collection, Processing, and Chemical Composition

The *Q. rugosa* CTWB used in this trial came from the collection reported by Carrillo-Muro et al. [19]. The species was identified at the Unidad Académica de Agronomía, UAZ, where the botanical samples were deposited in the herbarium. No collection permit was obtained. The material was collected using litter traps positioned approximately 0.30 m above ground level during the dry season and natural defoliation period, from 1 March to 30 April 2013, at Rancho Arroyo de Carrillo, Tequila, Jalisco, Mexico (21°15′07.30″ N, 103°39′48.12″ W; 1617 m above sea level). The traps intercepted naturally fallen plant material, which may have included bark and twigs in addition to leaves; the proportions of these components were not determined. This collection procedure limited direct contact with the soil, although soil contamination was not quantified. The collected biomass was ground in a hammer mill through a 25-mm screen and stored in laminated bags on pallets in a dry facility protected from light, moisture, and pests. The biomass was stored for approximately 7–9 months between collection and the beginning of the feeding trial. Its composition was not reanalyzed immediately before this trial; diet formulation therefore relied on the original characterization of the same collected biomass. Although storage conditions were intended to protect the material, compositional stability during storage was not verified.

In that study, representative CTWB samples were analyzed in triplicate. The DM content and ash were determined according to AOAC International [29]; CP was measured by Dumas combustion using an FP-528 nitrogen analyzer (LECO Corporation, St. Joseph, MI, USA); ether extract (EE) was determined with an ANKOM XT15 extractor (ANKOM Technology, Macedon, NY, USA) following method Am 5-04 [30]; and the NDF content was measured without α-amylase, while acid detergent fiber (ADF) was determined according to Van Soest et al. [31]. The CT concentration was determined using the butanol–HCl assay of Porter et al. [32]. Based on this characterization, the CTWB contained 85.0% DM and, on a DM basis, 3.9% CP, 1.5% EE, 58.2% NDF, 6.0% ash, and 2.62% CT. The previous characterization reported ADF concentrations of 49.11–50.30% on a DM basis across collection periods [19].

The CS was ground through a 25-mm screen, matching the screen size used for CTWB. Representative CS samples were analyzed in triplicate for DM, ash, CP, EE, and NDF using the procedures described above. Samples of the mixed diets were also analyzed for these components using the same procedures. The CS contained 89.0% DM and, on a DM basis, 6.5% CP, 2.1% EE, 65.0% NDF, and 7.2% ash. The CT concentrations were not quantified in CS or the mixed diets; accordingly, the reported dietary CT concentrations represent only the calculated contribution from CTWB, based on its analyzed concentration and proportion in each diet.

### 2.3. Animals, Housing, Experimental Design, and Diets

Forty-eight intact male Dorper × Katahdin lambs, with a mean initial age of 6 months (standard deviation: 7 d) and an initial body weight (IBW) of 33.01 ± 1.23 kg, were housed two per pen in 24 pens measuring 2.0 × 2.0 m. Each pen was equipped with a feeder and a waterer. Pens were assigned by simple randomization using a draw to four dietary treatments, with six pens per treatment and no stratification by IBW. The trial followed a completely randomized design, with the pen as the experimental unit. Before the trial, lambs were identified, dewormed by subcutaneous injection of 1% ivermectin (IVOMEC^®^; Boehringer Ingelheim Animal Health, Mexico City, Mexico) at 1 mL/50 kg body weight (BW; 0.2 mg/kg BW), and vaccinated subcutaneously against pneumonic pasteurellosis with 2 mL of One Shot^®^ (Zoetis México, Mexico City, Mexico). They were then adapted to the housing, management procedures, and experimental diets for 15 d. Water was available at all times during adaptation and the 42 d evaluation period.

The CTWB progressively replaced CS within a fibrous fraction held constant at 24% of dietary DM (Table 1). The CTWB-0, CTWB-8, CTWB-16, and CTWB-24 diets contained CS:CTWB proportions of 24:0, 16:8, 8:16, and 0:24%, respectively; all other ingredients remained constant. The calculated dietary CT contributions from CTWB were 0, 2.10, 4.19, and 6.29 g/kg DM, respectively. Diets were formulated on a DM basis to meet the requirements of finishing lambs established by NRC [33]. Ingredient proportions and dietary chemical composition are presented in Table 1.

Feed was offered at 09:00 and 16:00 h. Each morning, 30 min before the first feeding, refusals from each pen were collected, weighed, and recorded. Their DM content was also determined. These records were used to adjust the afternoon allotment and maintain refusals at approximately 30 g/kg of feed offered. The morning allotment remained at 500 g DM per lamb, equivalent to 1 kg DM per pen and approximately 40% of the anticipated daily intake.

### 2.4. Growth Performance

Lambs were weighed individually on 25 November 2013, before the first experimental feeding and after a 12 h fast from solid feed, to determine IBW. After 42 d of feeding, they were weighed again on the morning of slaughter, 6 January 2014, following an 18 h fast. This weight was used as both final body weight (FBW) and preslaughter body weight (PSBW). Weights were recorded with an electronic scale with a 200-kg capacity (CRS-200, Torrey, Monterrey, NL, Mexico). Cumulative BW change (CBWC) was calculated as FBW − IBW, and average daily gain (ADG) as (CBWC × 1000)/42, expressed in g/d. Both variables were averaged across the two lambs in each pen. Feed offered and refused were recorded daily by pen. Daily DMI per lamb was calculated as the difference between DM offered and refused in each pen, using the measured DM contents of the mixed diets and feed refusals. This difference was divided by two and averaged over the 42 d feeding period. The gain-to-feed ratio (G:F) was calculated by dividing the mean ADG of the two lambs, converted to kg/d, by the DMI per lamb in the same pen.

### 2.5. Ultrasound Measurements

Rib fat thickness (FAT, mm) and *longissimus* muscle area (LMA, cm^2^) were measured between the 12th and 13th ribs on the left side using real-time ultrasonography. Baseline measurements were obtained on day 0 after a 12 h fast from solid feed; final measurements were obtained on the morning of slaughter after an 18 h fast. Before each examination, wool was removed, the area was cleaned with compressed air, and acoustic gel was applied. Images were acquired with an Aloka ProSound 2 ultrasound system (Hitachi Aloka Medical, Tokyo, Japan) equipped with a 3.5-MHz linear transducer positioned perpendicular to the spine to visualize a cross-section of the *longissimus* muscle. Subcutaneous fat thickness over the muscle was measured as FAT, and LMA was determined by tracing the muscle’s cross-sectional outline. Both measurements were made directly on the images using the system’s built-in measurement tools. All images were acquired and evaluated by the same trained technician, who was blinded to dietary treatment.

### 2.6. Blood Metabolite Analyses

Blood samples (10 mL) were collected by jugular venipuncture on day 0 after a 12 h fast from solid feed and on the morning of slaughter after an 18 h fast. Both collections were performed at 07:00 h using evacuated tubes (BD Vacutainer^®^, Becton Dickinson, Franklin Lakes, NJ, USA); the baseline sample was obtained before feed was offered. Samples were centrifuged at 2500× *g* for 15 min to obtain serum, which was kept at 4 °C and analyzed on the day of collection. Baseline serum values were used as covariates for the corresponding final measurements.

Analyses were performed by spectrophotometry at the UAZ University Health Center using a Jenway 6320D spectrophotometer (Bibby Scientific Ltd., Staffordshire, UK) and commercial diagnostic kits (Diagnostic Chemicals Ltd., Charlottetown, PE, Canada) according to the manufacturer’s instructions. Serum concentrations, expressed in mg/dL, included glucose (GLU), triglycerides (TG), total cholesterol (TC), high-density lipoprotein cholesterol (HDL), very-low-density lipoprotein cholesterol (VLDL), low-density lipoprotein cholesterol (LDL), uric acid (UA), creatinine (CREA), and blood urea nitrogen (BUN). The BUN/CREA ratio was calculated from the corresponding serum concentrations.

### 2.7. Slaughter Procedures and Carcass Measurements

After the 42 d experimental period, lambs were fasted from feed for 18 h. The individual weight recorded on the morning of slaughter was used as PSBW. Following weighing, the final ultrasound examination, and blood collection, the lambs were transported approximately 0.5 km to the UAMVZ-UAZ abattoir, where they were slaughtered, skinned, and eviscerated. All 48 lambs were slaughtered on the same day under the same preslaughter and slaughter conditions. Individual weights were recorded for the skin, limbs, head, heart, lungs, liver, spleen, kidneys, testicles, omental fat, mesenteric fat, and perirenal fat. The stomach complex (rumen, reticulum, omasum, and abomasum), large intestine, and small intestine were weighed with their digesta contents. Visceral fat was calculated as the sum of omental and mesenteric fat; perirenal fat was evaluated separately. The stomach complex and intestines were then emptied and washed to determine their digesta-free weights. Total gastrointestinal digesta content was calculated as the difference between the full and empty weights of these components. Empty body weight (EBW) was calculated as PSBW minus total gastrointestinal digesta content, and non-carcass components were expressed as g/kg EBW.

After evisceration, the kidneys and internal fat were returned to the carcasses after individual weighing. Carcasses were then weighed immediately, before entering the chilling chamber, to determine hot carcass weight (HCW). They were chilled at 4 °C for 24 h before cold carcass weight (CCW) was recorded. Both HCW and CCW included the kidneys and internal fat. Both weights were measured using the same electronic scale (CRS-200, Torrey, Monterrey, NL, Mexico; capacity: 200 kg; resolution: 0.05 kg). Dressing percentage was calculated as (CCW/PSBW) × 100, and cooling loss (CL) as [(HCW − CCW)/HCW] × 100.

### 2.8. Wholesale Cuts and Tissue Composition

After chilling, the left carcass side was divided into forequarter and hindquarter, which were weighed before cutting. Following the guidelines of the North American Meat Processors Association [34], the neck, shoulder (Institutional Meat Purchase Specifications, IMPS, 206 and 207), rack (IMPS 204), breast (IMPS 209), and ribs (IMPS 209A) were obtained from the forequarter; the loin (IMPS 231), flank (IMPS 232), and leg (IMPS 233) were obtained from the hindquarter. The weight of each quarter was expressed as a percentage of the combined forequarter and hindquarter weights from the same animal; each individual cut from the left half-carcass was expressed as a percentage of whole-carcass CCW.

Shoulder (IMPS 207) tissue composition was determined by physical dissection according to Luaces et al. [35]. Muscle and fat were separated manually and weighed, and their proportions were expressed as percentages of the shoulder weight. The muscle-to-fat ratio was calculated from these components.

### 2.9. Meat-Quality Analyses

Samples of the *longissimus lumborum* muscle were collected from the left carcass side between the 12th rib and the second lumbar vertebra. Meat pH was measured 24 h postmortem (pH_24h_) using a portable pH meter equipped with a penetration electrode (Hanna Instruments HI-9025, Woonsocket, RI, USA).

Color was evaluated using a Minolta CR-400 colorimeter (Konica Minolta Sensing Inc., Osaka, Japan), calibrated beforehand with white and black standards. After 60 min of blooming, lightness (L*), redness (a*), and yellowness (b*) were recorded on the surface of the *longissimus lumborum* between the 12th and 13th ribs, following the Commission Internationale de l’Éclairage (CIE) L*a*b* system [36]. Kidney fat and dorsal fat color were evaluated using the same procedure.

The *longissimus lumborum* was cut into steaks, vacuum-packaged, and stored at −20 °C until analysis. Samples used for the water-loss and shear-force measurements described below were thawed at 4 °C for 24 h before their respective analyses.

Expressible water (EW) was determined using the modified Grau and Hamm method described by Tsai and Ockerman [37]. Approximately 0.3 g of muscle was placed between Whatman No. 1 filter papers and two glass plates, then subjected to a constant 10-kg load for 20 min. The EW percentage was calculated as [(final filter-paper weight − initial filter-paper weight)/initial sample weight] × 100. Results were expressed as a percentage of initial sample weight; higher values indicated greater water release under pressure.

To determine drip loss over 24 h (DL_24h_), thawed samples were blotted dry. One steak was cut into blocks of approximately 15 × 15 × 30 mm, which were weighed before being suspended at 4 °C for 24 h. The DL_24h_ percentage was calculated as [(initial weight − final weight)/initial weight] × 100. The initial weight was recorded after thawing; therefore, the measurement excluded exudate released during thawing and represented subsequent drip loss from previously frozen meat.

Cooking loss (CKL) was determined using a separate thawed steak placed in a sealed plastic bag and heated in a water bath at 80 °C until its internal temperature reached 70 °C. The sample was then blotted dry and weighed. The CKL percentage was calculated as [(weight before cooking − weight after cooking)/weight before cooking] × 100.

Warner–Bratzler shear force (WBSF) was determined according to the American Meat Science Association [38]. Thawed steaks 2.54 cm thick were cooked on an electric grill to an internal temperature of 70 °C. After cooking, they were allowed to cool at room temperature for 4 h. Six portions were prepared from each steak using the attachment supplied with the Warner–Bratzler device (G-R Manufacturing Co., Manhattan, KS, USA) and oriented so that the blade cut perpendicular to the muscle fibers. Peak shear force was recorded in kgf, and the mean of the six measurements from each steak was used for statistical analysis.

### 2.10. Economic Evaluation

The economic evaluation covered the 42-d experimental period and used DMI recorded by pen, individual CCW, and the cost of each diet. The DMI per lamb was obtained by dividing pen-level DMI by two. Ingredient and carcass prices reflected market conditions when the manuscript was prepared, rather than during the experimental period. The CTWB cost was updated by applying prices current at manuscript preparation to the collection labor, milling, sacks, and transport required for the amount of biomass collected. The CTWB was valued at 0.03450 USD/kg DM. Diet cost, calculated on a DM basis from ingredient proportions and unit prices, was 0.301, 0.295, 0.288, and 0.282 USD/kg DM for CTWB-0, CTWB-8, CTWB-16, and CTWB-24, respectively; carcasses were valued at 11.00 USD/kg CCW. For each lamb, total feed cost (USD/lamb) was calculated as DMI (kg DM/d) × 42 d × diet cost (USD/kg DM); carcass income (USD/lamb) as CCW (kg) × 11.00 USD/kg; income over feed cost (IOFC; USD/lamb) as carcass income − total feed cost; and cost of gain (USD/kg BW gained) as total feed cost/CBWC. Values for the two lambs were averaged by pen before statistical analysis.

### 2.11. Statistical Analysis

Data were analyzed using SAS^®^ OnDemand for Academics: SAS Studio, version 3.83 (SAS Institute Inc., Cary, NC, USA) [39]. Residual normality and homogeneity of variance were assessed using the Shapiro–Wilk and Levene tests, respectively. The trial was analyzed as a completely randomized design, with dietary treatment included as a fixed categorical effect to compare diets representing partial or complete replacement of CS with CTWB, and pen as the experimental unit for all outcomes, including growth performance, carcass characteristics, and meat quality. Individual measurements from the two lambs were averaged by pen before analysis; DMI and intake-derived variables were calculated from pen-level records. Variables analyzed without a covariate followed the model Y_ij_ = μ + T_i_ + ε_ij_, where Y_ij_ was the response for pen j receiving treatment i, μ was the overall mean, T_i_ was the treatment effect, and ε_ij_ was the residual error. For adjusted variables, the term β(X_ij_ − $\bar{X}$) was added, where β was the regression coefficient, X_ij_ was the corresponding mean baseline value for the pen, and $\bar{X}$ was the overall mean of that covariate. Pen-mean IBW was used as a covariate for FBW, and day-0 values of each blood metabolite were used as covariates for their respective final measurements obtained on the morning of slaughter. All other variables, including final FAT and LMA measurements and pen-mean economic indicators, were analyzed without a covariate. 

No *a priori* power calculation was performed, and no primary outcome was prespecified for sample size determination. Sample size was based on the availability of 24 pens, with six assigned to each of the four treatments; accordingly, the responses were interpreted as exploratory. Treatment means were compared using Tukey’s test within each variable, with no additional adjustment for multiplicity across different variables. Results are presented as least-squares means and standard errors of the mean (SEM), with the largest standard error of the treatment means reported for each variable. Mean differences between each CTWB-containing diet and CTWB-0 were estimated using the corresponding statistical model and are presented with individual 95% confidence intervals in Tables S1–S6. These intervals were calculated using the model-based standard errors of the differences and the corresponding residual degrees of freedom from the Student’s t distribution, without adjustment for multiple comparisons. Differences were considered significant at *p* ≤ 0.05.

Personnel who prepared and offered the diets knew the treatment assignments. The technicians responsible for ultrasound, carcass, and meat-quality evaluations were blinded to dietary treatment, and statistical analyses were performed using coded treatment labels.

## 3. Results

### 3.1. Growth Performance

Complete replacement of CS in the fibrous fraction with CTWB-24, which supplied a calculated 6.29 g CT/kg DM, increased DMI relative to CTWB-0 and CTWB-16 (1.34 vs. 1.17 and 1.18 kg DM/d, respectively; overall treatment effect, *p* = 0.021; Table 2). Intake with CTWB-8 was intermediate (1.20 kg DM/d). Despite the greater intake with CTWB-24, no significant treatment differences were detected in FBW, ADG, CBWC, or G:F (*p* ≥ 0.240). Mean differences from CTWB-0 with 95% confidence intervals are presented in Table S1.

### 3.2. Blood Metabolites

With complete replacement of CS, lambs fed CTWB-24 had lower BUN concentrations than those fed CTWB-8 (10.33 vs. 18.45 mg/dL; overall treatment effect: *p* = 0.014; Table 3). Values for CTWB-0 and CTWB-16 were intermediate. The BUN/CREA ratio, CREA, and UA did not differ among treatments (*p* ≥ 0.162). Neither did the lipid profile measurements (LDL, VLDL, HDL, TG, and TC) or GLU (*p* > 0.05). Mean differences from CTWB-0 with 95% confidence intervals are presented in Table S2.

### 3.3. Empty Body Weight, Non-Carcass Components, and Internal Fat Depots

Complete replacement of CS with CTWB-24 did not result in a difference in EBW relative to the other treatments (*p* = 0.391; Table 4). Relative weights of the stomach complex (*p* = 0.095), large intestine (*p* = 0.242), and small intestine (*p* = 0.732) did not differ, nor did those of the skin (*p* = 0.706), limbs (*p* = 0.991), and head (*p* = 0.318). Among the organs evaluated, the heart (*p* = 0.581), lungs (*p* = 0.940), liver (*p* = 0.251), spleen (*p* = 0.068), kidneys (*p* = 0.365), and testicles (*p* = 0.572) showed no differences among treatments. The same was observed for omental (*p* = 0.247), mesenteric (*p* = 0.206), and perirenal (*p* = 0.675) fat depots and for calculated visceral fat (*p* = 0.236). Mean differences from CTWB-0 with 95% confidence intervals are presented in Table S3.

### 3.4. Ultrasound Traits, Carcass Characteristics, and Shoulder Tissue Composition

Neither CL nor dressing percentage differed among treatments (*p* = 0.459 and *p* = 0.060, respectively; Table 5). Likewise, HCW and CCW did not differ among treatments (*p* ≥ 0.779). Rib FAT and LMA also did not differ (*p* ≥ 0.668). No differences were observed in forequarter and hindquarter proportions or in individual wholesale cut proportions, including the leg (*p* > 0.05), or in shoulder muscle and fat proportions or the muscle-to-fat ratio (*p* ≥ 0.394). Mean differences from CTWB-0 with 95% confidence intervals are presented in Table S4.

### 3.5. Meat Quality

Complete replacement of CS with CTWB-24 did not result in differences from CTWB-0 in pH_24h_, DL_24h_, EW, CKL, or WBSF; these measurements also did not differ among the other treatments (*p* ≥ 0.103; Table 6). An overall treatment effect was observed for *longissimus lumborum* a* (*p* = 0.049), but Tukey’s test did not identify differences between individual treatments. Dorsal fat b* did not differ among treatments (*p* = 0.051). The L* and b* coordinates of the *longissimus lumborum*, all three color coordinates of kidney fat, and the L* and a* coordinates of dorsal fat did not differ among treatments (*p* > 0.05). Mean differences from CTWB-0 with 95% confidence intervals are presented in Table S5.

### 3.6. Economic Evaluation

Complete replacement of CS with CTWB-24 reduced calculated diet cost from 0.301 to 0.282 USD/kg DM relative to CTWB-0 (Table 7). Although DMI was greater with CTWB-24, total feed cost did not differ among treatments (15.87 vs. 14.81 USD/lamb for CTWB-24 and CTWB-0, respectively; *p* = 0.150). Carcass income, IOFC, and cost of gain also did not differ (*p* ≥ 0.578). The IOFC was 205.56, 200.87, 203.78, and 202.21 USD/lamb for CTWB-0, CTWB-8, CTWB-16, and CTWB-24, respectively (*p* = 0.895). Cost of gain was 1.98, 1.78, 1.92, and 1.76 USD/kg BW gained, respectively (*p* = 0.578). Thus, under the prices evaluated, complete replacement of CS lowered calculated diet cost per kilogram of DM, while total feed cost and cost of gain did not differ among treatments. Mean differences from CTWB-0 with 95% confidence intervals are presented in Table S6.

## 4. Discussion

### 4.1. Growth Performance

Complete replacement of CS with 24% CTWB increased DMI relative to CTWB-0 and CTWB-16, but no significant treatment differences were detected in ADG or G:F over 42 d. Greater intake without a demonstrated growth advantage is relevant to the practical assessment of this replacement, although it does not establish reduced feed efficiency. Replacement simultaneously changed the fiber source and diet composition and supplied a calculated contribution of 6.29 g CT/kg DM; consequently, the responses cannot be attributed specifically to CT. Previous studies illustrate the variability of responses to tannin-containing feeds [7,8], including reduced intake or growth with carob pulp and oak foliage [11,17] and differing intake and growth responses with extracts [13,40,41]. These comparisons provide background, but differences in ingredients, species, and basal diets limit their explanatory value for CTWB.

A more directly relevant ingredient comparison is provided by Carrillo-Muro et al. [24], who evaluated *Q. rugosa* biomass in Rambouillet lambs intended for breeding. With a 60% forage fraction over 70 d, 20% CTWB supported greater ADG and G:F than 60% CTWB, whereas DMI did not differ among treatments. This comparison highlights that complete CS replacement can involve substantially different biomass inclusion levels, but does not establish a common response across production stages or diets. In the present trial, the greater DMI with 24% CTWB remains unexplained because digestibility and ruminal measurements were not obtained. The nonsignificant ADG and G:F results do not demonstrate equivalence, and the limited replication does not exclude biologically relevant differences.

### 4.2. Blood Metabolites

No significant treatment differences were detected in the lipid profile or GLU. Studies with tannin-containing diets have likewise reported unchanged TC [42] or metabolic responses that varied with the basal diet [43]. However, these comparisons do not explain the response to replacing CS with CTWB, because the ingredients and dietary conditions differed.

The BUN difference was restricted to CTWB-24 versus CTWB-8; neither differed significantly from CTWB-0. It therefore does not establish a progressive decline with increasing inclusion and warrants caution given the exploratory analysis of multiple outcomes. Changes in N-related blood measurements have also been reported with other oak foliage [17,44,45], but these findings do not demonstrate a common mechanism. The lower dietary CP concentration accompanying CS replacement is a possible contributor to the BUN response, although its role was not isolated. Neither CREA nor BUN/CREA differed significantly among diets. Without measurements of digestibility, ruminal N metabolism, or N balance, the BUN finding cannot establish improved N utilization or a determining role for CT.

### 4.3. Empty Body Weight, Non-Carcass Components, and Internal Fat Depots

The greater DMI with CTWB-24 was not accompanied by significant treatment differences in EBW or the relative weights of the gastrointestinal components and liver. Dietary forage characteristics and energy intake can influence visceral tissue mass in lambs [46,47], but the present measurements do not identify how these tissues responded functionally to CS replacement. Comparisons with changes reported in steers receiving mimosa or chestnut extracts [48] are limited because extract supplementation differs from replacing the fibrous ingredient with woody biomass.

No significant treatment differences were detected in internal fat depots. Nevertheless, visceral fat was numerically higher with CTWB-16 and CTWB-24 than with CTWB-0, although the values did not increase consistently with inclusion level. These numerical differences do not establish a treatment effect or altered nutrient partitioning.

### 4.4. Ultrasound Traits, Carcass Characteristics, and Shoulder Tissue Composition

Greater DMI with CTWB-24 did not establish an advantage in HCW or CCW, and no significant treatment differences were detected in final FAT or LMA. Limited responses in ultrasound traits or carcass weight have also been reported with other tannin-containing diets [14,40,49]. Conversely, lower carcass weights accompanied reduced intake and growth in some studies [11,13]. These comparisons provide context, but cannot explain the response to replacing CS with CTWB because the ingredients and diets differed.

Neither CL nor dressing percentage differed significantly among treatments. The numerical reduction in CL was not accompanied by greater CCW and therefore does not demonstrate a commercial benefit in carcass weight. Cooling loss represents whole-carcass weight loss during chilling, distinct from the water losses measured subsequently in muscle samples; agreement between these measurements should not be assumed.

No significant treatment differences were detected in wholesale cut proportions or shoulder tissue composition, consistent with the limited shoulder response reported by Rojas-Román et al. [14]. Nevertheless, shoulder fat declined numerically, and the muscle-to-fat ratio increased across inclusion levels. These numerical patterns do not establish a treatment effect or altered tissue partitioning.

### 4.5. Meat Quality

No significant treatment differences were detected in pH_24h_, most muscle color coordinates, or fat color. Muscle a* showed an overall treatment effect, but Tukey’s test did not identify differences between individual diets; this finding therefore does not establish a specific replacement level associated with altered redness. Dorsal fat b* was nonsignificant. Variable pH and color responses have been reported with other tannin-containing feeds [10,11,41,50], but differences in ingredients and measurement conditions limit their relevance to CTWB. Color was assessed after 60 min of blooming, without repeated measurements during storage or lipid oxidation measurements, so these results cannot establish color stability or antioxidant protection.

No significant treatment differences were detected in DL_24h_, EW, CKL, or WBSF. Limited responses in water loss and shear force have also been reported with other tannin-containing diets [42,49,51], whereas greater shear force has been observed with some extracts [10,52]. In the present study, DL_24h_ was measured during the 24 h period following thawing and initial weighing, excluding exudate released during thawing. Accordingly, the water-loss and shear-force findings are restricted to the previously frozen and thawed samples evaluated and cannot be extrapolated to fresh meat.

### 4.6. Economic Evaluation

Complete replacement of CS with CTWB lowered calculated diet cost from 0.301 to 0.282 USD/kg DM, but the greater DMI offset this saving: total feed cost was numerically higher with CTWB-24 than with CTWB-0 (15.87 vs. 14.81 USD/lamb). Cost of gain was numerically lower (1.76 vs. 1.98 USD/kg BW gained), but neither indicator differed significantly among treatments. Differences from the control and their 95% confidence intervals are presented in Table S6. Thus, lower diet cost per kilogram did not establish an economic advantage per lamb.

No CTWB inclusion level demonstrated an IOFC advantage over CTWB-0, and nonsignificant results do not establish economic equivalence. Practical assessment therefore requires considering intake, growth, and carcass income together with biomass availability and local prices. The evaluation used prices current at manuscript preparation, including an updated CTWB cost of 0.03450 USD/kg DM; its applicability would need reassessment if ingredient costs or carcass value changed. The CTWB cost was calculated from collection labor, milling, sacks, and transport expenses relative to the amount of biomass collected, and expressed per kilogram of DM. These costs may vary among regions and production scales; therefore, the value used here is specific to the collection and processing conditions evaluated.

Beyond price, the amount of biomass required determines the potential scale of replacement. At the observed DMI with CTWB-24, each lamb consumed approximately 13.5 kg CTWB DM over 42 d. The maximum litter accumulation of 616 kg DM/ha reported by Carrillo-Muro et al. [19] would theoretically supply approximately 46 lambs per hectare for this period, assuming complete recovery and use. Actual supply would depend on seasonal and site variation, collection and storage losses, and the proportion of litter retained for ecological functions. This calculation provides an estimate of land requirements, rather than evidence of sustained commercial availability.

### 4.7. Study Limitations

Replacing CS with CTWB simultaneously changed the fiber source, dietary CP and NDF concentrations, and calculated CT contribution. The design evaluated the whole ingredient and could not isolate CT effects; these compounds remain potential contributors rather than established determinants of the responses. The CT were not quantified in CS or the mixed diets, so the reported concentrations represent only the calculated contribution from CTWB. Stored biomass was not reanalyzed before feeding. Therefore, its nutrient and CT concentrations at feeding were not verified, and storage-related changes in CT and other secondary compounds cannot be excluded. The calculated CT contribution may consequently differ from the concentration actually consumed. Future large-scale trials should reanalyze the biomass before feeding and monitor potential changes in tannins and other secondary compounds during postharvest handling and storage. Characterization also lacked lignin, hydrolysable tannins, total phenols, particle-size distribution, and physically effective NDF; the milling screen size alone does not establish fiber physical effectiveness. The butanol–HCl calibration standard was not specified in the original report [19], further limiting interpretation of the CT concentration.

Digestibility, ruminal fermentation, microbial protein synthesis, ruminal N metabolism, and N balance were not measured. Consequently, the processes underlying greater DMI and the BUN difference remain unresolved. Baseline and final measurements followed 12 and 18 h of feed withdrawal, respectively. Although fasting duration was identical across treatments at each sampling, the different durations limit baseline-to-final comparability for BW change, ADG, and blood metabolites. Diet-related differences in residual gastrointestinal fill and their contribution to the responses could not be quantified.

Meat pH was measured only at 24 h postmortem, and color after 60 min of blooming, without storage-time measurements. Lipid oxidation stability, fatty acid composition, antioxidant status, and sensory characteristics were not evaluated. Thus, the results do not establish color stability, antioxidant protection, or effects on fatty acid composition or sensory quality. Water-loss and shear-force findings are also restricted to the previously frozen and thawed samples evaluated.

The exploratory analysis of multiple outcomes and limited pen replication warrant caution, particularly for isolated significant findings. Nonsignificant results do not establish equivalence or exclude biologically relevant differences. Finally, the economic evaluation used prices current at manuscript preparation, including the updated, collection- and processing-specific CTWB cost, without a sensitivity analysis. These limitations restrict mechanistic interpretation and extrapolation to other diets, feeding periods, production conditions, or price scenarios.

## 5. Conclusions

Under the conditions of this study, a diet containing 24% CTWB from *Q. rugosa* was evaluated with no CS in the fibrous fraction during a 42-d finishing period. Although DMI increased, no significant treatment differences were detected in ADG, G:F, HCW, CCW, the principal meat-quality measurements, or the economic indicators. These exploratory findings describe the response to complete dietary replacement of CS under the conditions evaluated, without establishing equivalence among diets or an economic advantage. With six pens per treatment, biologically relevant differences cannot be ruled out.

## Figures and Tables

**Table 1:** Ingredient proportions and analyzed and calculated chemical composition of the experimental diets.

Ingredients, % DM	Treatments ^1^			
	CTWB-0	CTWB-8	CTWB-16	CTWB-24
Corn stover	24.00	16.00	8.00	0.00
Condensed tannin-containing woody biomass	0.00	8.00	16.00	24.00
Steam-flaked corn	48.00	48.00	48.00	48.00
Soybean meal	12.00	12.00	12.00	12.00
Molasses, cane	8.00	8.00	8.00	8.00
Vegetable fat	3.50	3.50	3.50	3.50
Sodium bentonite	1.25	1.25	1.25	1.25
Calcium carbonate	1.10	1.10	1.10	1.10
Sodium sesquicarbonate	1.00	1.00	1.00	1.00
Salt	0.25	0.25	0.25	0.25
Urea	0.80	0.80	0.80	0.80
Premix ^2^	0.10	0.10	0.10	0.10
**Chemical composition, % ^3^**				
Dry matter	87.22	86.90	86.58	86.26
Organic matter	93.19	93.28	93.38	93.47
Crude protein	14.96	14.75	14.54	14.33
Neutral detergent fiber	21.71	21.16	20.62	20.08
Ether extract	6.23	6.18	6.13	6.09
Ash	6.81	6.72	6.62	6.53
Calcium	0.74	0.69	0.65	0.60
Phosphorus	0.27	0.26	0.25	0.24
Calcium/Phosphorus ratio	2.80	2.68	2.56	2.44
Condensed tannins, g/kg DM	0.00	2.10	4.19	6.29

^1^ CTWB-0, CTWB-8, CTWB-16, and CTWB-24 denote diets containing 0, 8, 16, and 24% condensed tannin-containing woody biomass (CTWB) on a dry matter (DM) basis, respectively. ^2^ Each kg of premix supplied 221.6 mg Co, 125,420 mg Fe, 402.5 mg I, 10,064 mg Mn, 17,078.5 mg Zn, 267 mg Se, 7813 mg Cu, 2,595,000 IU of vitamin A, 468,750 IU of vitamin D, and 28,000 IU of vitamin E. ^3^ Dry matter, ash, crude protein, ether extract, and neutral detergent fiber were determined in the mixed diets. Dry matter is given on an as-fed basis and the other components on a DM basis. Organic matter equals 100 − ash. Calcium (Ca) and phosphorus (P) were calculated from tabulated values for all ingredients except CTWB [33]. The contribution from CTWB was excluded because its Ca and P concentrations were unavailable; the reported values therefore do not represent total dietary Ca and P concentrations. Dietary condensed tannin (CT) concentrations represent the calculated contribution from CTWB; CT were not quantified in corn stover or the mixed diets.

**Table 2:** Growth performance in finishing lambs fed condensed tannin-containing woody biomass (CTWB; 48 lambs, 24 pens, two lambs per pen, six pens per treatment) at increasing levels.

Item ^1^	Treatments ^2^				SEM ^3^	*p*-Value
	CTWB-0	CTWB-8	CTWB-16	CTWB-24		
Body weight, kg						
Initial	33.25	32.78	32.98	33.02	0.65	0.939
Final	41.44	41.72	40.68	42.77	0.78	0.258
ADG, g/d	199.80	208.53	182.74	232.44	18.31	0.240
Cumulative body weight change, kg	8.39	8.76	7.68	9.76	0.77	0.240
DMI, kg DM/d	1.17 ^b^	1.20 ^ab^	1.18 ^b^	1.34 ^a^	0.04	0.021
Gain-to-feed ratio	0.168	0.174	0.155	0.173	0.01	0.489

^1^ ADG = average daily gain; DMI = dry-matter intake; DM = dry matter. ^2^ CTWB-0, CTWB-8, CTWB-16, and CTWB-24 denote diets containing 0, 8, 16, and 24% CTWB on a dry-matter basis, respectively. ^3^ SEM = standard error of the mean. Values are presented as least-squares means. ^a,b^ Means within a row with different superscripts differ (*p* ≤ 0.05) according to Tukey’s multiple comparison test.

**Table 3:** Blood metabolites in finishing lambs fed condensed tannin-containing woody biomass (CTWB; 48 lambs, 24 pens, two lambs per pen, six pens per treatment) at increasing levels.

Item ^1^	Treatments ^2^				SEM ^3^	*p*-Value
	CTWB-0	CTWB-8	CTWB-16	CTWB-24		
LDL, mg/dL	14.63	16.46	15.80	16.55	1.90	0.883
VLDL, mg/dL	4.02	8.51	1.98	2.23	1.87	0.083
HDL, mg/dL	32.04	29.66	30.95	27.23	5.34	0.912
GLU, mg/dL	73.67	74.46	83.87	80.34	4.76	0.411
TG, mg/dL	43.78	42.38	52.76	75.75	11.50	0.221
TC, mg/dL	49.57	56.43	54.25	68.42	9.16	0.532
BUN, mg/dL	15.54 ^ab^	18.45 ^a^	15.76 ^ab^	10.33 ^b^	1.28	0.014
BUN/CREA	20.29	26.19	16.98	11.87	4.32	0.162
CREA, mg/dL	0.80	0.71	1.19	0.88	0.29	0.565
UA, mg/dL	0.95	0.66	0.91	1.53	0.24	0.168

^1^ LDL = low-density lipoprotein cholesterol; VLDL = very-low-density lipoprotein cholesterol; HDL = high-density lipoprotein cholesterol; GLU = glucose; TG = triglycerides; TC = total cholesterol; BUN = blood urea nitrogen; CREA = creatinine; BUN/CREA = blood urea nitrogen-to-creatinine ratio; UA = uric acid. ^2^ CTWB-0, CTWB-8, CTWB-16, and CTWB-24 denote diets containing 0, 8, 16, and 24% CTWB on a dry-matter basis, respectively. ^3^ SEM = standard error of the mean. Values are presented as least-squares means. ^a,b^ Means within a row with different superscripts differ (*p* ≤ 0.05) according to Tukey’s multiple comparison test.

**Table 4:** Empty body weight, non-carcass components, and internal fat depots in finishing lambs fed condensed tannin-containing woody biomass (CTWB; 48 lambs, 24 pens, two lambs per pen, six pens per treatment) at increasing levels.

Item	Treatments ^1^				SEM ^2^	*p*-Value
	CTWB-0	CTWB-8	CTWB-16	CTWB-24		
Empty body weight, kg	36.94	36.23	35.80	37.13	0.60	0.391
**Non-carcass components and internal fat depots, g/kg empty body weight**						
Stomach complex	32.87	35.38	32.91	35.97	1.04	0.095
Large intestine	14.34	15.62	15.65	17.72	1.14	0.242
Small intestine	22.52	23.45	25.11	23.31	1.66	0.732
Skin	161.02	162.78	169.39	169.35	6.37	0.706
Limbs	18.58	18.41	18.36	18.50	0.51	0.991
Head	41.56	44.49	43.98	41.36	1.45	0.318
Heart	6.92	5.99	5.57	5.72	0.74	0.581
Lungs	27.25	26.27	25.62	26.53	1.86	0.940
Liver	23.19	23.11	21.71	23.15	0.59	0.251
Spleen	2.60	2.23	2.43	2.13	0.13	0.068
Kidneys	3.10	3.25	3.34	3.32	0.11	0.365
Testicles	11.17	10.88	11.62	12.67	0.95	0.572
Omental fat	18.64	17.68	22.04	22.47	1.97	0.247
Mesenteric fat	12.14	11.26	11.88	13.36	0.68	0.206
Visceral fat	30.78	28.93	33.92	35.83	2.49	0.236
Perirenal fat	7.03	7.73	9.18	8.80	1.47	0.675

^1^ CTWB-0, CTWB-8, CTWB-16, and CTWB-24 denote diets containing 0, 8, 16, and 24% CTWB on a dry-matter basis, respectively. ^2^ SEM = standard error of the mean. Values are presented as least-squares means.

**Table 5:** Ultrasound traits, carcass characteristics, quarter and wholesale cut proportions, and shoulder tissue composition in finishing lambs fed condensed tannin-containing woody biomass (CTWB; 48 lambs, 24 pens, two lambs per pen, six pens per treatment) at increasing levels.

Item ^1^	Treatments ^2^				SEM ^3^	*p*-Value
	CTWB-0	CTWB-8	CTWB-16	CTWB-24		
**Ultrasound traits**						
FAT, mm	3.24	3.32	3.24	3.25	0.15	0.968
LMA, cm^2^	13.09	12.72	12.85	12.35	0.47	0.668
**Carcass characteristics**						
HCW, kg	20.88	20.26	20.48	20.70	0.44	0.779
CCW, kg	20.03	19.61	19.83	20.00	0.41	0.874
Dressing percentage, %	47.53	47.20	48.25	45.95	0.56	0.060
Cooling loss, %	4.02	3.21	3.20	3.36	0.41	0.459
**Forequarter and hindquarter, % of their combined weight**						
Forequarter	57.77	55.88	57.03	57.73	1.29	0.657
Hindquarter	42.23	44.12	42.97	42.27	1.29	0.657
**Left-side wholesale cuts, % of whole-carcass CCW**						
Neck	4.54	3.98	4.35	4.54	0.48	0.784
Shoulder IMPS 206	8.12	8.12	7.92	7.72	0.27	0.666
Shoulder IMPS 207	6.07	6.13	5.88	5.89	0.39	0.941
Rack IMPS 204	2.56	2.80	2.25	2.51	0.20	0.224
Breast IMPS 209	4.65	4.62	4.41	4.83	0.20	0.493
Ribs IMPS 209A	3.01	2.37	2.87	2.88	0.18	0.082
Loin IMPS 231	3.94	3.89	3.76	3.06	0.80	0.844
Flank IMPS 232	3.19	2.75	3.08	3.15	0.26	0.569
Leg IMPS 233	14.04	15.48	13.99	14.59	0.41	0.053
**Shoulder (IMPS 207) tissue composition, %**						
Muscle	64.53	64.51	66.05	65.98	1.23	0.615
Fat	14.87	14.04	13.13	11.56	1.51	0.434
Muscle:fat ratio	4.39	4.85	5.19	5.95	0.67	0.394

^1^ FAT = rib fat thickness; LMA = longissimus muscle area; HCW = hot carcass weight; CCW = cold carcass weight; IMPS = Institutional Meat Purchase Specifications. ^2^ CTWB-0, CTWB-8, CTWB-16, and CTWB-24 denote diets containing 0, 8, 16, and 24% CTWB on a dry-matter basis, respectively. ^3^ SEM = standard error of the mean. Values are presented as least-squares means.

**Table 6:** Meat quality and muscle and fat color in finishing lambs fed condensed tannin-containing woody biomass (CTWB; 48 lambs, 24 pens, two lambs per pen, six pens per treatment) at increasing levels.

Item ^1^	Treatments ^2^				SEM ^3^	*p*-Value
	CTWB-0	CTWB-8	CTWB-16	CTWB-24		
**Meat-quality characteristics**						
DL_24h_, %	1.91	2.30	3.20	1.95	0.70	0.491
CKL, %	16.86	16.76	19.57	20.86	1.40	0.165
Expressible water, %	36.63	36.77	34.86	34.54	0.78	0.103
WBSF, kgf	3.98	4.37	3.98	4.22	0.35	0.828
***Longissimus lumborum* color and pH**						
pH_24h_	5.77	5.79	5.85	5.78	0.04	0.601
L*	39.37	42.17	39.14	40.73	0.93	0.111
a*	18.42	15.96	18.40	18.31	0.68	0.049
b*	5.70	4.59	6.48	5.86	0.54	0.128
**Kidney fat color**						
L*	68.81	69.86	69.86	69.02	1.11	0.864
a*	12.41	11.57	13.23	13.83	0.74	0.188
b*	11.53	10.68	12.31	12.30	0.52	0.114
**Dorsal fat color**						
L*	69.62	67.26	68.33	67.86	0.94	0.360
a*	9.09	8.71	9.20	10.70	0.72	0.255
b*	14.02	12.82	14.36	15.31	0.59	0.051

^1^ DL_24h_ = drip loss during 24 h of suspension at 4 °C in previously frozen and thawed samples; CKL = cooking loss; WBSF = Warner–Bratzler shear force; pH_24h_ = pH measured 24 h postmortem; L* = lightness; a* = redness; b* = yellowness. ^2^ CTWB-0, CTWB-8, CTWB-16, and CTWB-24 denote diets containing 0, 8, 16, and 24% CTWB on a dry-matter basis, respectively. ^3^ SEM = standard error of the mean. Values are presented as least-squares means.

**Table 7:** Economic evaluation of finishing lambs fed condensed tannin-containing woody biomass (CTWB; 48 lambs, 24 pens, two lambs per pen, six pens per treatment) at increasing levels.

Item ^1^	Treatments ^2^				SEM ^3^	*p*-Value
	CTWB-0	CTWB-8	CTWB-16	CTWB-24		
Calculated diet cost, USD/kg DM ^4^	0.301	0.295	0.288	0.282	—	—
Total feed cost, USD/lamb	14.81	14.82	14.29	15.87	0.50	0.150
Carcass income, USD/lamb	220.37	215.69	218.08	218.08	5.71	0.917
IOFC, USD/lamb	205.56	200.87	203.78	202.21	5.46	0.895
Cost of gain, USD/kg BW gained	1.98	1.78	1.92	1.76	0.15	0.578

^1^ Economic outcomes were calculated for each lamb and averaged within pen before analysis; pen was the experimental unit. DM = dry matter; USD = United States dollar; BW = body weight; IOFC = income over feed cost. A dash (—) indicates that no inferential comparison was performed. Mean differences from CTWB-0 with 95% confidence intervals are presented in Table S6. ^2^ CTWB-0, CTWB-8, CTWB-16, and CTWB-24 denote diets containing 0, 8, 16, and 24% CTWB on a dry-matter basis, respectively. ^3^ SEM = standard error of the mean. Values are presented as least-squares means. ^4^ Ingredient and carcass prices corresponded to manuscript preparation rather than the experimental period. The CTWB cost was updated using collection labor, milling, sacks, and transport costs expressed per kilogram of DM collected.

## Data Availability

The data presented in this study are available from the corresponding author upon reasonable request.

## Supplementary Materials

The following supporting information can be downloaded at: https://www.mdpi.com/article/10.3390/ani16213339/s1. Table S1: Mean differences from CTWB-0 with 95% confidence intervals for growth performance and feed efficiency in finishing lambs; Table S2: Mean differences from CTWB-0 with 95% confidence intervals for blood metabolites in finishing lambs; Table S3: Mean differences from CTWB-0 with 95% confidence intervals for empty body weight and non-carcass components in finishing lambs; Table S4: Mean differences from CTWB-0 with 95% confidence intervals for ultrasound traits, carcass characteristics, wholesale cuts, and shoulder tissue composition in finishing lambs; Table S5: Mean differences from CTWB-0 with 95% confidence intervals for meat quality and muscle and fat color in finishing lambs; Table S6: Mean differences from CTWB-0 with 95% confidence intervals for economic outcomes in finishing lambs.
