# Supplementary material for: Corn Stover Replacement with Quercus rugosa Woody Biomass: Growth Performance, Carcass Characteristics, and Meat Quality of Finishing Lambs
Source: Animals (Basel). 2026 Oct 23;16(21):3339. doi: 10.3390/ani16213339 (PMC13761161; doi:10.3390/ani16213339)
Supplement: Supplementary file 1 [file animals-16-03339-s001.zip › animals-4624326-supplementary.pdf]

Supplementary Materials

Corn Stover Replacement with *Quercus rugosa* Woody Biomass: Growth Performance, Carcass Characteristics, and Meat Quality of Finishing Lambs

Table S1. Mean differences from CTWB-0 with 95% confidence intervals for growth performance and feed efficiency in finishing lambs.

Item	CTWB-8 − CTWB-0	CTWB-16 − CTWB-0	CTWB-24 − CTWB-0
Initial body weight, kg	−0.48<br>(−2.06, 1.11)	−0.27<br>(−1.85, 1.32)	−0.23<br>(−2.00, 1.55)
Final body weight, kg	0.28<br>(−1.64, 2.21)	−0.76<br>(−2.68, 1.15)	1.33<br>(−0.80, 3.46)
ADG, g/d	8.73<br>(−35.68, 53.14)	−17.06<br>(−61.47, 27.35)	32.64<br>(−17.01, 82.29)
Cumulative body weight change, kg	0.37<br>(−1.50, 2.23)	−0.72<br>(−2.58, 1.15)	1.37<br>(−0.71, 3.46)
DMI, kg DM/d	0.02<br>(−0.07, 0.12)	0.01<br>(−0.09, 0.11)	0.17<br>(0.06, 0.28)
G:F	0.006<br>(−0.022, 0.034)	−0.014<br>(−0.042, 0.015)	0.004<br>(−0.027, 0.036)

[1] Values are model-based mean differences (diet − CTWB-0), with individual 95% confidence intervals in parentheses. Confidence intervals are not adjusted for multiple comparisons and do not replace the Tukey comparisons reported in the main tables. Intervals use model-based standard errors and residual degrees of freedom. CTWB = condensed tannin-containing woody biomass. Final body weight was adjusted for initial body weight. ADG = average daily gain; DMI = dry matter intake; DM = dry matter; G:F = gain-to-feed ratio.

Table S2. Mean differences from CTWB-0 with 95% confidence intervals for blood metabolites in finishing lambs.

**Item**	**CTWB-8 − CTWB-0**	**CTWB-16 − CTWB-0**	**CTWB-24 − CTWB-0**
LDL, mg/dL	1.83 (−4.49, 8.15)	1.16 (−5.17, 7.50)	1.91 (−4.50, 8.33)
VLDL, mg/dL	4.49 (−1.39, 10.38)	−2.04 (−8.56, 4.48)	−1.79 (−9.85, 6.28)
HDL, mg/dL	−2.38 (−23.11, 18.36)	−1.08 (−17.37, 15.20)	−4.81 (−23.66, 14.05)
GLU, mg/dL	0.79 (−14.70, 16.28)	10.20 (−4.79, 25.19)	6.67 (−8.33, 21.67)
TG, mg/dL	−1.40 (−39.74, 36.94)	8.98 (−26.85, 44.82)	31.97 (−3.64, 67.59)
TC, mg/dL	6.86 (−23.69, 37.42)	4.67 (−25.11, 34.46)	18.84 (−10.90, 48.58)
BUN, mg/dL	2.91 (−1.47, 7.29)	0.22 (−3.95, 4.40)	−5.21 (−9.37, −1.04)
BUN/CREA	5.90 (−8.70, 20.49)	−3.31 (−17.23, 10.60)	−8.42 (−22.92, 6.08)
CREA, mg/dL	−0.08 (−0.87, 0.70)	0.39 (−0.44, 1.22)	0.08 (−0.92, 1.08)
UA, mg/dL	−0.29 (−0.98, 0.39)	−0.03 (−0.80, 0.73)	0.58 (−0.20, 1.36)

Values are model-based mean differences (diet − CTWB-0), with individual 95% confidence intervals in parentheses. Confidence intervals are not adjusted for multiple comparisons and do not replace the Tukey comparisons reported in the main tables. Intervals use model-based standard errors and residual degrees of freedom. CTWB = condensed tannin-containing woody biomass. Differences were adjusted for the corresponding baseline metabolite measurement. BUN = blood urea nitrogen; CREA = creatinine; GLU = glucose; TG = triglycerides; TC = total cholesterol; UA = uric acid; LDL, VLDL, and HDL = low-, very-low-, and high-density lipoprotein cholesterol.

Table S3. Mean differences from CTWB-0 with 95% confidence intervals for empty body weight and non-carcass components in finishing lambs.

Item	CTWB-8 − CTWB-0	CTWB-16 − CTWB-0	CTWB-24 − CTWB-0
Empty body weight, kg	−0.72<br>(−2.50, 1.06)	−1.14<br>(−2.92, 0.64)	0.18<br>(−1.60, 1.97)
Stomach complex, g/kg EBW	2.50<br>(−0.57, 5.58)	0.03<br>(−3.04, 3.11)	3.10<br>(0.02, 6.18)
Large intestine, g/kg EBW	1.28<br>(−2.07, 4.63)	1.30<br>(−2.05, 4.66)	3.38<br>(0.02, 6.73)
Small intestine, g/kg EBW	0.93<br>(−3.96, 5.82)	2.59<br>(−2.29, 7.48)	0.79<br>(−4.09, 5.68)
Skin, g/kg EBW	1.75<br>(−17.05, 20.56)	8.37<br>(−10.43, 27.18)	8.33<br>(−10.48, 27.13)
Limbs, g/kg EBW	−0.17<br>(−1.67, 1.33)	−0.21<br>(−1.71, 1.29)	−0.07<br>(−1.57, 1.42)
Head, g/kg EBW	2.93<br>(−1.34, 7.19)	2.42<br>(−1.85, 6.68)	−0.20<br>(−4.46, 4.06)
Heart, g/kg EBW	−0.93<br>(−3.12, 1.25)	−1.35<br>(−3.53, 0.83)	−1.20<br>(−3.39, 0.98)
Lungs, g/kg EBW	−0.98<br>(−6.45, 4.49)	−1.62<br>(−7.10, 3.85)	−0.72<br>(−6.19, 4.76)
Liver, g/kg EBW	−0.08<br>(−1.83, 1.67)	−1.47<br>(−3.22, 0.27)	−0.03<br>(−1.78, 1.71)
Spleen, g/kg EBW	−0.37<br>(−0.74, 0.01)	−0.17<br>(−0.54, 0.20)	−0.47<br>(−0.84, −0.10)
Kidneys, g/kg EBW	0.14<br>(−0.17, 0.45)	0.24<br>(−0.07, 0.55)	0.22<br>(−0.09, 0.53)
Testicles, g/kg EBW	−0.29<br>(−3.08, 2.51)	0.45<br>(−2.35, 3.25)	1.50<br>(−1.30, 4.30)
Omental fat, g/kg EBW	−0.97<br>(−6.77, 4.84)	3.40<br>(−2.41, 9.21)	3.83<br>(−1.98, 9.63)
Mesenteric fat, g/kg EBW	−0.88<br>(−2.89, 1.13)	−0.26<br>(−2.27, 1.75)	1.22<br>(−0.79, 3.23)
Visceral fat, g/kg EBW	−1.85<br>(−9.21, 5.51)	3.14<br>(−4.22, 10.50)	5.05<br>(−2.31, 12.41)
Perirenal fat, g/kg EBW	0.70<br>(−3.59, 4.99)	2.15<br>(−2.14, 6.44)	1.77<br>(−2.52, 6.07)

Values are model-based mean differences (diet − CTWB-0), with individual 95% confidence intervals in parentheses. Confidence intervals are not adjusted for multiple comparisons and do not replace the Tukey comparisons reported in the main tables. Intervals use model-based standard errors and residual degrees of freedom. CTWB = condensed tannin-containing woody biomass. EBW = empty body weight.

Table S4. Mean differences from CTWB-0 with 95% confidence intervals for ultrasound traits, carcass characteristics, wholesale cuts, and shoulder tissue composition in finishing lambs.

Item	CTWB-8 − CTWB-0	CTWB-16 − CTWB-0	CTWB-24 − CTWB-0
FAT, mm	0.08<br>(−0.30, 0.45)	0.00<br>(−0.37, 0.37)	0.01<br>(−0.41, 0.42)
LMA, cm^2	−0.38<br>(−1.51, 0.76)	−0.24<br>(−1.37, 0.90)	−0.75<br>(−2.01, 0.52)
Hot carcass weight, kg	−0.62<br>(−1.92, 0.68)	−0.40<br>(−1.70, 0.91)	−0.18<br>(−1.48, 1.12)
Cold carcass weight, kg	−0.43<br>(−1.62, 0.77)	−0.21<br>(−1.41, 0.99)	−0.03<br>(−1.23, 1.16)
Dressing percentage, %	−0.33<br>(−1.99, 1.33)	0.72<br>(−0.94, 2.38)	−1.57<br>(−3.23, 0.09)
Cooling loss, %	−0.81<br>(−2.02, 0.40)	−0.82<br>(−2.03, 0.39)	−0.66<br>(−1.87, 0.55)
Forequarter[3], %	−1.88<br>(−5.70, 1.93)	−0.73<br>(−4.55, 3.08)	−0.03<br>(−4.11, 4.04)
Hindquarter[3], %	1.88<br>(−1.93, 5.70)	0.73<br>(−3.08, 4.55)	0.03<br>(−4.04, 4.11)
Neck[1], %	−0.56<br>(−1.97, 0.86)	−0.20<br>(−1.61, 1.22)	0.00<br>(−1.51, 1.51)
Shoulder IMPS 206[1], %	0.00<br>(−0.80, 0.79)	−0.20<br>(−0.99, 0.59)	−0.40<br>(−1.25, 0.45)
Shoulder IMPS 207[1], %	0.07<br>(−1.09, 1.23)	−0.19<br>(−1.35, 0.97)	−0.17<br>(−1.41, 1.07)
Rack IMPS 204[1], %	0.24<br>(−0.34, 0.82)	−0.31<br>(−0.89, 0.27)	−0.05<br>(−0.67, 0.57)
Breast IMPS 209[1], %	−0.03<br>(−0.62, 0.56)	−0.24<br>(−0.83, 0.35)	0.18<br>(−0.45, 0.81)
Ribs IMPS 209A[1], %	−0.63<br>(−1.16, −0.10)	−0.14<br>(−0.67, 0.39)	−0.13<br>(−0.70, 0.44)
Loin IMPS 231[1], %	−0.05<br>(−2.41, 2.31)	−0.18<br>(−2.54, 2.18)	−0.88<br>(−3.40, 1.64)
Flank IMPS 232[1], %	−0.44<br>(−1.22, 0.34)	−0.11<br>(−0.89, 0.67)	−0.04<br>(−0.87, 0.79)
Leg IMPS 233[1], %	1.44<br>(0.23, 2.64)	−0.05<br>(−1.26, 1.16)	0.54<br>(−0.75, 1.83)
Shoulder muscle[2], %	−0.01<br>(−3.59, 3.57)	1.53<br>(−1.62, 4.67)	1.46<br>(−2.12, 5.03)
Shoulder fat[2], %	−0.83<br>(−5.23, 3.57)	−1.74<br>(−5.60, 2.13)	−3.31<br>(−7.71, 1.09)
Shoulder muscle:fat ratio	0.47<br>(−1.49, 2.42)	0.80<br>(−0.91, 2.52)	1.57<br>(−0.39, 3.52)

Values are model-based mean differences (diet − CTWB-0), with individual 95% confidence intervals in parentheses. Confidence intervals are not adjusted for multiple comparisons and do not replace the Tukey comparisons reported in the main tables. Intervals use model-based standard errors and residual degrees of freedom. CTWB = condensed tannin-containing woody biomass. FAT = rib fat thickness; LMA = longissimus muscle area. Final FAT and LMA were analyzed without a covariate. [1] Left-side wholesale cuts expressed as percentages of whole-carcass cold carcass weight. [2] Tissue proportions expressed as percentages of shoulder weight. IMPS = Institutional Meat Purchase Specifications. [3] Forequarter and hindquarter were expressed as percentages of their combined weight for each lamb and then averaged by pen.

Table S5. Mean differences from CTWB-0 with 95% confidence intervals for meat quality and muscle and fat color in finishing lambs.

Item	CTWB-8 − CTWB-0	CTWB-16 − CTWB-0	CTWB-24 − CTWB-0
DL_{24h}[1], %	0.39<br>(−1.63, 2.41)	1.29<br>(−0.73, 3.31)	0.04<br>(−2.09, 2.17)
Cooking loss, %	−0.10<br>(−4.19, 4.00)	2.71<br>(−1.38, 6.81)	4.00<br>(−0.66, 8.66)
Expressible water, %	0.14<br>(−2.09, 2.38)	−1.76<br>(−4.00, 0.47)	−2.09<br>(−4.44, 0.27)
Warner–Bratzler shear force, kgf	0.39<br>(−0.69, 1.48)	0.01<br>(−1.08, 1.09)	0.25<br>(−0.84, 1.33)
pH at 24 h postmortem	0.02<br>(−0.12, 0.15)	0.08<br>(−0.06, 0.21)	0.00<br>(−0.13, 0.13)
Muscle L*	2.80<br>(0.06, 5.54)	−0.23<br>(−2.97, 2.51)	1.36<br>(−1.38, 4.10)
Muscle a*	−2.45<br>(−4.47, −0.43)	−0.01<br>(−2.03, 2.00)	−0.11<br>(−2.13, 1.91)
Muscle b*	−1.11<br>(−2.71, 0.48)	0.78<br>(−0.81, 2.38)	0.15<br>(−1.44, 1.75)
Kidney fat L*	1.04<br>(−2.24, 4.32)	1.05<br>(−2.23, 4.32)	0.20<br>(−3.07, 3.48)
Kidney fat a*	−0.84<br>(−3.03, 1.35)	0.82<br>(−1.37, 3.01)	1.43<br>(−0.76, 3.62)
Kidney fat b*	−0.86<br>(−2.38, 0.67)	0.77<br>(−0.75, 2.30)	0.77<br>(−0.76, 2.30)
Dorsal fat L*	−2.36<br>(−5.13, 0.42)	−1.28<br>(−4.06, 1.49)	−1.75<br>(−4.53, 1.02)
Dorsal fat a*	−0.38<br>(−2.51, 1.76)	0.11<br>(−2.02, 2.25)	1.61<br>(−0.52, 3.75)
Dorsal fat b*	−1.21<br>(−2.94, 0.52)	0.33<br>(−1.40, 2.07)	1.29<br>(−0.45, 3.02)

Values are model-based mean differences (diet − CTWB-0), with individual 95% confidence intervals in parentheses. Confidence intervals are not adjusted for multiple comparisons and do not replace the Tukey comparisons reported in the main tables. Intervals use model-based standard errors and residual degrees of freedom. CTWB = condensed tannin-containing woody biomass. [1] DL_{24h} = drip loss over 24 h, measured during 24 h of suspension at 4 °C in previously frozen and thawed samples. L*, a*, and b* represent lightness, redness, and yellowness, respectively.

Table S6. Mean differences from CTWB-0 with 95% confidence intervals for economic outcomes in finishing lambs.

Item	CTWB-8 − CTWB-0	CTWB-16 − CTWB-0	CTWB-24 − CTWB-0
Total feed cost, USD/lamb	0.01<br>(−1.20, 1.23)	−0.52<br>(−1.73, 0.70)	1.06<br>(−0.30, 2.42)
Carcass income, USD/lamb	−4.68<br>(−18.54, 9.19)	−2.29<br>(−16.15, 11.57)	−2.29<br>(−17.79, 13.21)
IOFC, USD/lamb	−4.69<br>(−17.94, 8.57)	−1.78<br>(−15.03, 11.48)	−3.35<br>(−18.17, 11.47)
Cost of gain, USD/kg BW gained	−0.21<br>(−0.58, 0.17)	−0.06<br>(−0.43, 0.31)	−0.22<br>(−0.64, 0.20)

Values are model-based mean differences (diet − CTWB-0), with individual 95% confidence intervals in parentheses. Confidence intervals are not adjusted for multiple comparisons and do not replace the Tukey comparisons reported in the main tables. Intervals use model-based standard errors and residual degrees of freedom. CTWB = condensed tannin-containing woody biomass. Diet costs were fixed at 0.301, 0.295, 0.288, and 0.282 USD/kg DM for CTWB-0, CTWB-8, CTWB-16, and CTWB-24, respectively; carcass value was 11.00 USD/kg cold carcass weight. Outcomes were calculated for each lamb and averaged by pen; the same pen records were used for all four economic outcomes. IOFC = income over feed cost; USD = United States dollar; BW = body weight; DM = dry matter.
